# Organization of Chemotherapy and Systemic Therapy Services in India: The Devil Lies in the Details

**DOI:** 10.1200/JGO.2016.006742

**Published:** 2016-10-12

**Authors:** Sameer Rastogi, Aditi Aggarwal

**Affiliations:** **Sameer Rastogi**, All India Institute of Medical Sciences, New Delhi; **Aditi Aggarwal**, Fortis Noida, Uttar Pradesh, India

## To the Editor:

The recent article by Gulia et al^1^ in *Journal of Global Oncology* proposed a model for chemotherapy delivery in India that effectively uses district health centers, medical colleges, and apex hospitals in hierarchical chemotherapy delivery according to the complexity of treatment and ease of administration. We believe that this model, although ideal, is more of theoretical interest because previously, a three-tier system for rural health miserably failed in India.^2^ As reported recently by Sharma et al,^3^ in community health centers (ie, the third tier in the rural health system) a huge deficit of physician-surgeons (83%), obstetricians and gynecologists (76%), physicians (83%), and pediatricians (82%) exists. These shocking statistics provoke ire among health professionals but still have not drawn any intervention or immediate actions by the government. Although these authors have proposed involvement of district hospitals, this seems difficult for various reasons. First, district hospitals and community health centers do not deliver the services they are supposed to, and the current quality of care is pathetic, with almost nonexistent services for basic medicine, surgery, gynecology, and pediatrics. In a nationally representative spatial analysis, Dare et al^4^ showed that approximately two thirds of deaths from acute abdominal conditions in India could have been averted by improvement in human and physical resources at existing district hospitals. The addition of medical oncology would be a burden too difficult to manage in the absence of basic resources. The lack of trained specialists in district hospitals is not because of the lack of trained professionals but because of the lack of government incentives, as reported recently in a district hospital in Meerut (second-tier city in India) where all specialist positions in the district hospital are vacant.^5^ Second, the administration of various modalities in different hospitals (eg, chemotherapy in district hospitals and radiation therapy in higher-volume centers) would be difficult on patients. Third, hardly any chemotherapy regimen in district hospital practice currently meets the criteria specified by Gulia et al^1^ because most of the regimens for breast cancer, lung cancer, and head and neck cancer cannot be administered in district hospitals for various reasons, such as extravasation and the need for premedication, hydration, and concurrent radiotherapy.^6^ Furthermore, patients might also prefer to go to specialized centers with expertise in multimodality treatment. Thus, before embarking on low-quality, erratic services in district hospitals, we believe it better to strengthen the already-crumbling health system of medical colleges and apex centers in India.

Even if this model or its modification is implemented, some ideas need clarification, first and foremost of which is the difficulties in coordination among all levels of care. In other words, the first step in implementing such a system is the improvement of outdated and virtually nonexistent information technology. Such improvements would help to track patient history and make quick referrals for various modalities and complications. This type of integrated health system would avoid duplication of duties and use personnel and the infrastructure optimally. Besides this, the largely unregulated private sector should be made accountable to use information technology and to properly document treatments given and cost regulation. As of now, the government sector caters only to a fraction of patients, and the majority of patients are treated in the private sector, so it is imperative to improve both systems simultaneously because the two are intertwined. Another thing that is of immense concern in this hierarchical system is the treatment of complex diseases, like sarcoma. There is plenty of literature that suggests that patients with sarcoma treated at higher-volume centers have better outcomes as compared to those treated at low-volume centres.^6^ Thus, we believe that it is not only the difficulty and administration of chemotherapy that governs the choice of center, but also the type of disease and complexity of diagnosis. Beyond this model, we believe that the government of India has confused priorities, as shown by the dip in the overall health budget,^7^ but yet the funds allotment to the ministry of ayurveda, yoga and naturopathy, unani, siddha, and homeopathy (traditional Indian medicines) was increased.^8^ Although we appreciate the earnest effort by Gulia et,^1^ we believe that the implementation of this model is difficult and impractical. However, we concede that such a model may be a harbinger for the evolution of the ideal model. Furthermore, the government of India should wake up to the call of the formidable opponent known as cancer and take immediate initiatives for the larger good of its people.
